# Quantitative analysis of blooming artifact caused by calcification based on X-ray energy difference using computed tomography

**DOI:** 10.1038/s41598-024-61187-z

**Published:** 2024-05-21

**Authors:** Daebeom Park, Eun-Ah Park, Baren Jeong, Yoon Seong Lee, Whal Lee

**Affiliations:** 1 Department of Radiology, Seoul National University Hospital, 101 Daehak-ro, Jongno-gu, Seoul, 03080 Korea; 2 Department of Radiology, Seoul National University College of Medicine, Seoul, Korea; 3 Institute of Radiation Medicine, Seoul National University Medical Research Center, Seoul, Korea; 4 Department of Clinical Medical Sciences, Seoul National University College of Medicine, Seoul, Korea

**Keywords:** Cardiovascular diseases, Medical imaging, Calcium and vitamin D, Cardiovascular biology

## Abstract

Blooming artifacts caused by calcifications appearing on computed tomography (CT) images lead to an underestimation of the coronary artery lumen size, and higher X-ray energy levels are suggested to reduce the blooming artifacts with subjective visual assessment. This study aimed to evaluate the effect of higher X-ray energy levels on the quantitative measurement of adjacent pixels affected by calcification using CT images. In this two-part study, CT images were acquired from dual-energy CT scanners by changing the X-ray energy levels such as kilovoltage peak (kVp) and kilo-electron volts (keV). Adjacent pixels affected by calcification were measured using the brightened length, excluding the actual calcified length, as determined by the full width at third maximum. In a separate clinical study, the adjacent affected pixels associated with 23 calcifications across 10 patients were measured using the same method as that used in the phantom study. Phantom and clinical studies showed that the change in kVp (field of view [FOV] 300 mm: p = 0.167, 0.494, and 0.861 for vendors 1, 2, and 3, respectively) and keV levels (p = 0.178 for vendor 2) failed to reduce the adjacent pixels affected by calcification, respectively. Moreover, the change in keV levels showed different aspects of adjacent pixels affected by calcification in the phantom study (FOV 300 mm: no significant difference [p = 0.191], increase [p < 0.001], and decrease [p < 0.001] for vendors 1, 2, and 3, respectively). Quantitative measurements revealed no significant relationship between higher X-ray energy levels and the adjacent pixels affected by calcification.

## Introduction

Coronary artery disease (CAD) is a major cause of death worldwide and a serious health problem that can pose various risks to the heart and blood vessels^1–3^. Therefore, many studies have focused on the treatment and prevention of CAD^1^. Computed tomography (CT) is widely used for noninvasive evaluation of CAD^4–7^. Conventional CT uses a polychromatic beam consisting of a wide spectrum of photon energies to reconstruct polychromatic images^8^. In contrast to conventional CT, dual-energy CT (DECT) utilizes multiple X-ray beams to synthesize a virtual monochromatic image from the obtained polychromatic images^9–12^. This technique offers an advantage in the dual absorption or emission of photons using different energy levels, enabling the decomposition of tissues into distinct components^9–12^. However, blooming artifacts can be observed in CT images of patient with high levels of coronary artery calcium when compared with coronary angiography images^13–16^. This situation leads to an underestimation of the actual vessel size when evaluating the stenosis degree of coronary arteries^13–16^.

Previous studies have been conducted to identify strategies to reduce blooming artifacts caused by calcification on CT images and have suggested increasing X-ray energy levels, such as the kilovoltage peak (kVp) in conventional polychromatic images or kilo-electron volts (keV) in virtual monochromatic images, with subjective visual assessment^17–20^. Higher X-ray energy levels decrease the attenuation values of calcification in the CT images, which can also be produced by increasing the width of the window. We hypothesized that higher X-ray energy levels decrease blooming artifacts caused by calcification with subjective visual assessment in CT images but fail to reduce adjacent pixels affected by calcification with quantitative measurement. This study was conducted to quantitatively analyze the relationship between different X-ray energy levels and adjacent pixels affected by calcification using phantom and clinical studies.

## Results

### Phantom study: acquirement of reconstructed computed tomography images

**Figure 1:**
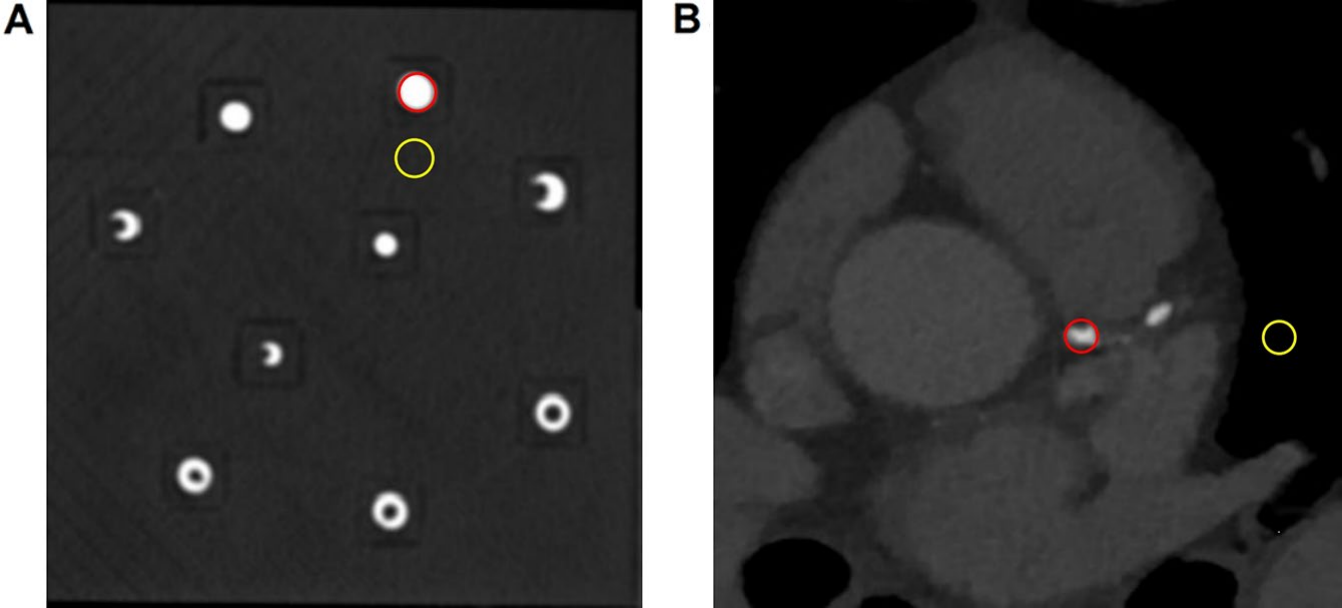
Reconstructed computed tomography images of a phantom and a patient. Reconstructed computed tomography image of a phantom (**A**) and a patient (**B**). Red and yellow lines indicate the region of interest (ROI) and the background region, respectively. Contrast to noise ratio, signal to noise ratio, and noise were calculated using the selected ROI and the background region.

To quantitatively investigate the relationship between adjacent pixels affected by calcification and different X-ray energy levels, such as kVp or keV, using various vendors, polychromatic and virtual monochromatic CT images of a phantom were acquired (Fig. 1). The distortion angle of the phantom was measured to accurately calculate the adjacent pixels affected by calcification. The results showed a maximum distortion angle of 2.06° (mean distortion angle = 1.27°, SD = 0.37) and a maximum measurement error of 0.06% (mean measurement error = 0.03%, SD = 0.01). Because of the low measurement error in the distortion angle of the phantom, reconstructed images with axial positions were used for the analysis of adjacent pixels affected by calcification (Table 1 and Supplementary Fig. S1).

**Table 1:** Distortion angles of CT images using a phantom.

		Scan 1	Scan 2	Scan 3	Scan 4	Scan 5
Distortion angle (degrees)	Vendor 1	1.56	1.40	1.01	1.40	1.16
	Vendor 2	1.42	2.06	1.23	0.76	1.22
	Vendor 3	1.03	1.87	1.32	0.92	0.75
Measurement error (%)	Vendor 1	0.04	0.03	0.02	0.03	0.02
	Vendor 2	0.03	0.06	0.02	0.01	0.02
	Vendor 3	0.02	0.05	0.03	0.01	0.01

Distortion angle and measurement error of CT images obtained from five repetitive scans for each vendor.

Vendor 1, Siemens; Vendor 2, Philips; Vendor 3, GE.

### Computed tomography image quality validation

Moreover, CT image quality was validated through contrast-to-noise ratio (CNR), signal-to-noise ratio (SNR), and noise (Fig. 1). Each value represented the quality of the reconstructed images obtained from each DECT scanner (Table 2). The CNR, SNR, and noise showed no significant differences among the kVp levels in the polychromatic images (p = 0.615, 0.801, and 0.801 for CNR, SNR, and noise, respectively). However, CNR and SNR, excluding noise, showed significant differences among keV levels in virtual monochromatic images owing to the exponential decrease in the maximum value of Hounsfield unit (HU) at higher keV levels (p = 0.003, 0.003, and 0.085 for CNR, SNR, and noise, respectively). Importantly, the maximum HU of calcification was significantly different among the kVp (p = 0.029) and keV (p = 0.003) levels with decreasing attenuation values at higher X-ray energy levels.

**Table 2:** Contrast-to-noise ratio, signal-to-noise ratio, and noise calculated for polychromatic and virtual monochromatic images using each vendor.

	Image type	X-ray energy	Computed tomography quality parameters			
			CNR	SNR	Noise	Maximum HU
Vendor 1	Polychromatic	80	95.10 ± 4.91	93.57 ± 4.90	12.10 ± 0.59	2276 ± 24
		100	85.81 ± 3.49	84.71 ± 3.38	10.94 ± 0.42	1873 ± 19
		120	79.10 ± 4.19	78.79 ± 4.11	10.41 ± 0.48	1645 ± 18
		140	82.23 ± 5.31	81.82 ± 5.27	9.17 ± 0.50	1505 ± 15
	Virtual monochromatic-phantom	40	110.43 ± 5.39	108.81 ± 5.33	15.41 ± 0.80	3071 ± 0
		70	96.68 ± 4.65	95.44 ± 4.59	7.33 ± 0.28	1404 ± 13
		100	56.09 ± 2.64	55.34 ± 2.62	7.73 ± 0.35	871 ± 9
		130	41.40 ± 2.04	40.83 ± 1.98	8.14 ± 0.41	687 ± 8
		140	38.84 ± 1.98	38.30 ± 1.90	8.23 ± 0.42	654 ± 8
Vendor 2	Polychromatic	80	122.87 ± 5.21	121.61 ± 5.18	9.72 ± 0.36	2404 ± 5
		100	109.11 ± 4.96	109.01 ± 4.96	9.05 ± 0.33	1994 ± 6
		120	87.86 ± 2.97	88.36 ± 2.98	9.75 ± 0.27	1744 ± 6
		140	77.52 ± 3.27	78.41 ± 3.29	10.00 ± 0.40	1568 ± 5
	Virtual monochromatic-phantom	40	203.22 ± 3.52	199.38 ± 3.23	8.80 ± 0.69	3071 ± 0
		70	115.81 ± 5.40	117.46 ± 5.44	6.40 ± 0.31	1493 ± 12
		100	74.60 ± 3.54	78.00 ± 3.68	6.21 ± 0.30	954 ± 10
		130	60.07 ± 3.04	64.07 ± 3.22	6.17 ± 0.32	772 ± 9
		140	57.57 ± 2.98	61.69 ± 3.18	6.15 ± 0.33	739 ± 9
	Virtual monochromatic-human	40	85.38 ± 12.68	49.97 ± 8.56	43.91 ± 9.98	2114 ± 147
		70	77.51 ± 9.66	26.77 ± 3.98	31.26 ± 7.74	912 ± 71
		100	68.94 ± 7.91	17.01 ± 2.42	29.29 ± 7.22	607 ± 47
		130	65.84 ± 7.42	13.59 ± 1.91	28.76 ± 7.05	504 ± 39
		140	65.16 ± 7.31	12.94 ± 1.81	28.68 ± 7.02	485 ± 37
Vendor 3	Polychromatic	80	54.68 ± 2.31	53.09 ± 2.29	18.01 ± 0.63	1976 ± 8
		100	62.15 ± 1.77	60.73 ± 1.80	12.90 ± 0.24	1617 ± 6
		120	64.66 ± 3.84	63.39 ± 3.83	11.05 ± 0.46	1418 ± 6
		140	63.05 ± 2.50	61.87 ± 2.63	10.12 ± 0.26	1274 ± 7
	Virtual monochromatic-phantom	40	71.45 ± 3.21	69.43 ± 3.11	24.54 ± 0.93	3071 ± 0
		70	65.51 ± 3.11	64.52 ± 3.03	11.69 ± 0.45	1498 ± 11
		100	56.27 ± 2.69	56.08 ± 2.64	8.39 ± 0.32	946 ± 6
		130	50.86 ± 2.42	51.11 ± 2.39	7.29 ± 0.28	758 ± 5
		140	49.62 ± 2.33	49.98 ± 2.31	7.08 ± 0.26	723 ± 5
P-value	Polychromatic	kVp levels	0.615	0.801	0.801	0.029
	Virtual monochromatic	keV levels	0.003	0.003	0.085	0.003

Data are represented as mean ± S.E.M of results. Friedman test was used for the calculation of p-value to compare the quality of computed tomography images among different X-ray energy levels.

CNR, contrast-to-noise ratio; HU, Hounsfield unit; keV, kilo-electron volts; kVp, kilovoltage peak; SNR, signal-to-noise ratio; Vendor 1, Siemens; Vendor 2, Philips; Vendor 3, GE.

### Subjective visual assessment of blooming artifact

**Figure 2:**
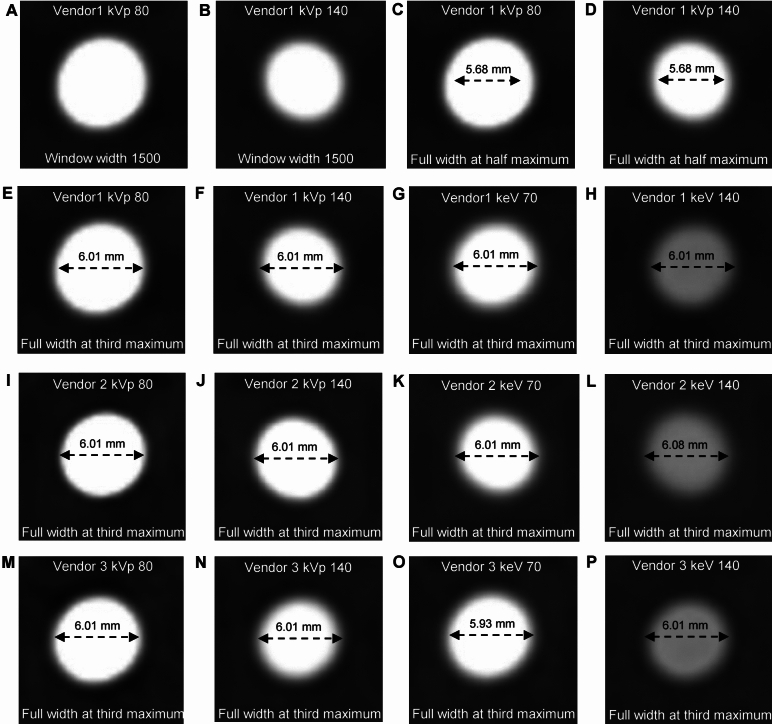
Computed tomography images of a phantom using different X-ray energy levels and vendors, along with quantitative measurements. Images of a concentric shape of calcification with a diameter of 6 mm, window center of 500, and width of 1500. (**A**, **B**) Different kilovoltage peak levels of 80 (**A**) and 140 (**B**) showed the reduced blooming artifacts based on subjective visual assessment. (**C**–**P**) Diameter calculated using the full width at half maximum (**C** and **D**) or the full width at third maximum (**E**–**P**), utilizing polychromatic and virtual monochromatic images from vendor 1, 2, and 3, obtained from representative computed tomography images of the phantom. Each image provides information regarding the vendor and image type. Vendor 1, Siemens; Vendor 2, Philips; Vendor 3, GE.

After validating the CT quality, blooming artifacts in the CT images were subjectively observed using different kVp levels through visual assessment. Higher kVp levels decreased the attenuation values of calcification, resulting in a reduction in blooming artifacts on subjective visual assessment (Fig. 2A and B). This finding demonstrates the dependency of subjective visual assessment of blooming artifacts on the X-ray energy levels of the CT images.

### Quantitative measurement of adjacent pixels affected by calcification

**Figure 3:**
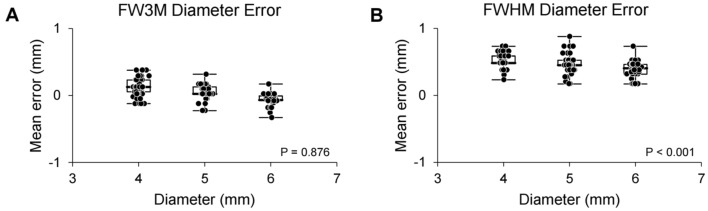
Box plots of diameter error when using full width at third or half maximum. (**A**, **B**) Box plots of diameter error of full width at third maximum (**A**) or full width at half maximum (**B**) using different sizes of calcification with concentric shape. The bold horizontal line indicates the median, while the top and bottom horizontal lines of boxes show the quartiles of 25% and 75%, respectively. Upper and lower horizontal lines outside of boxes show the maximum and minimum, respectively. Wilcoxon signed-rank test was used for the calculation of p-value comparing with the reference error of 0.04 mm. FWHM, full width at half maximum; FW3M, full width at third maximum.

After the subjective visual assessment of blooming artifacts, adjacent pixels affected by calcification were quantitatively measured for reconstructed images using each DECT vendor to validate the relationship with higher or lower X-ray energy levels. Adjacent pixels affected by calcification was calculated by subtracting the brightened length from the actual calcified length (Supplementary Fig. S2). To determine the actual calcified length, the full width at half maximum (FWHM) was measured for each kVp or keV level and showed consistency regardless of the different attenuation values from calcification (Fig. 2C and D). However, the full width at third maximum (FW3M) measured the actual calcified length with a mean error less than that of the FWHM when using different kVp or keV levels from various vendors (Fig. 2E–P). Moreover, the mean error using FW3M showed no significant difference when compared with the reference error of 0.04 mm (mean error = 0.05 mm, S.E.M = 0.01 mm, and p = 0.876) (Fig. 3A). Mean error for measuring the actual calcified length using FWHM was 0.47 mm and showed significant difference when compared with the reference error of 0.04 mm (mean error = 0.47 mm, S.E.M = 0.01 mm, and p < 0.001) (Fig. 3B). Adjacent pixels affected by calcification were quantitatively analyzed using the brightened length, excluding the actual calcified length determined by the FW3M, in both phantom and clinical studies, considering various parameters of each DECT vendor.

### Phantom study: analysis of adjacent pixels affected by calcification using polychromatic images

**Figure 4:**
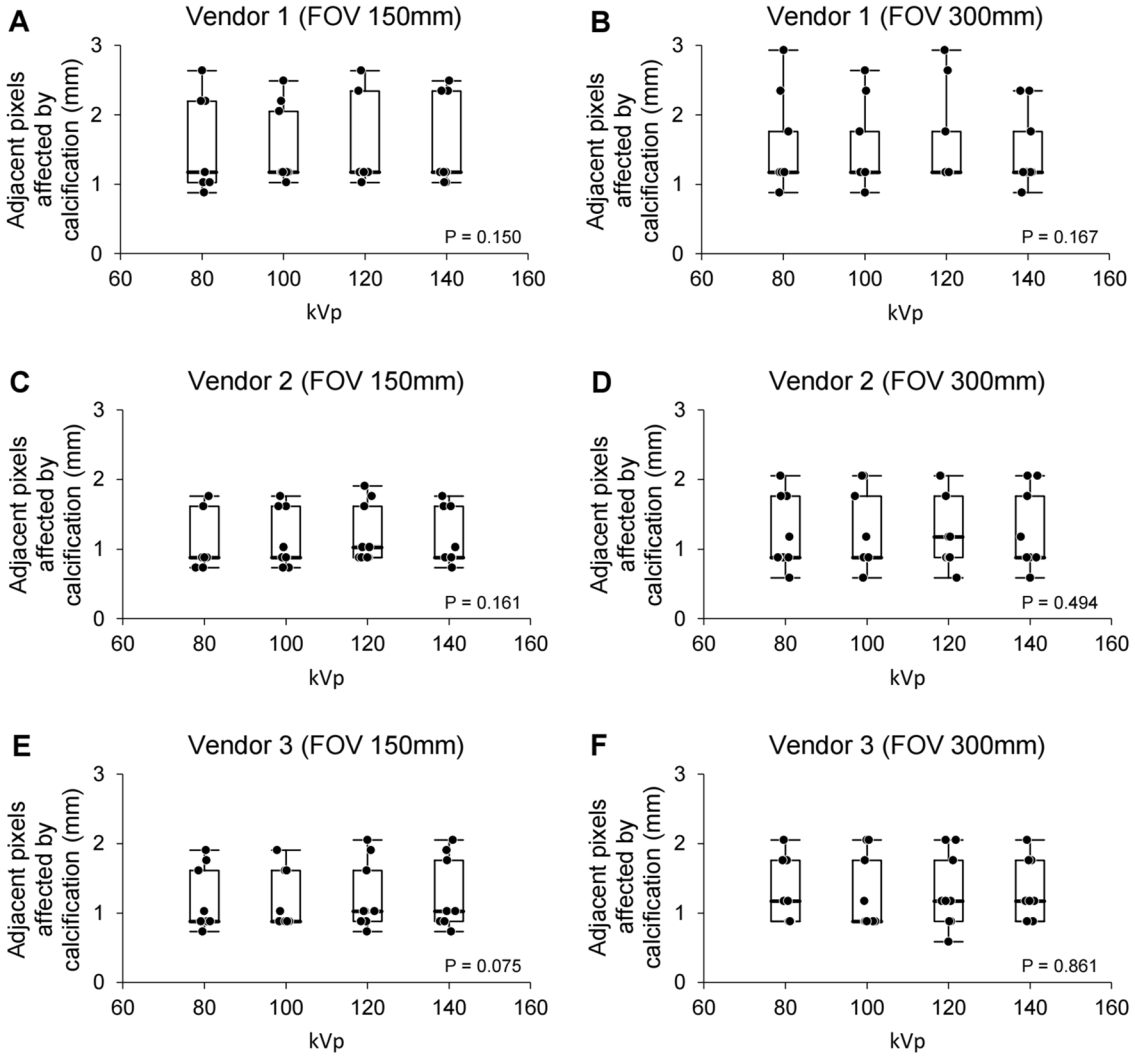
Box plots of adjacent pixels affected by calcification using different kilovoltage peak levels of polychromatic images of a phantom. Box plots of adjacent pixels affected by calcification for different kilovoltage peak levels for small (**A**, **C**, and **E**) or large (**B**, **D**, and **F**) field of view of polychromatic images of a phantom using vendor 1 (**A** and **B**), 2 (**C** and **D**), or 3 (**E** and **F**). The bold horizontal line indicates the median, while the top and bottom horizontal lines of boxes show the quartiles of 25% and 75%, respectively. Upper and lower horizontal lines outside of boxes show the maximum and minimum, respectively. Friedman test was used for the calculation of p-value. FOV, field of view; kVp, kilovoltage peak; Vendor 1, Siemens; Vendor 2, Philips; Vendor 3, GE.

First, adjacent pixels affected by calcification were calculated using polychromatic images with different kVp levels. Using polychromatic images, adjacent pixels affected by calcification were compared for each kVp level (Fig. 4), and the results failed to show significant differences in adjacent pixels affected by calcification among the kVp levels in both small and large field of view (FOV) images for each CT vendor (FOV 300 mm, p = 0.167, 0.494 and 0.861 for vendors 1, 2, and 3, respectively; FOV 150 mm, p = 0.150, 0.161, and 0.075 for vendors 1, 2, and 3, respectively). Quantitative analysis using polychromatic images indicated that the low kVp level with a lower radiation dose showed a similar quantitative measurement of adjacent pixels affected by calcification compared to high kVp levels.

### Phantom study: analysis of adjacent pixels affected by calcification using virtual monochromatic images

**Figure 5:**
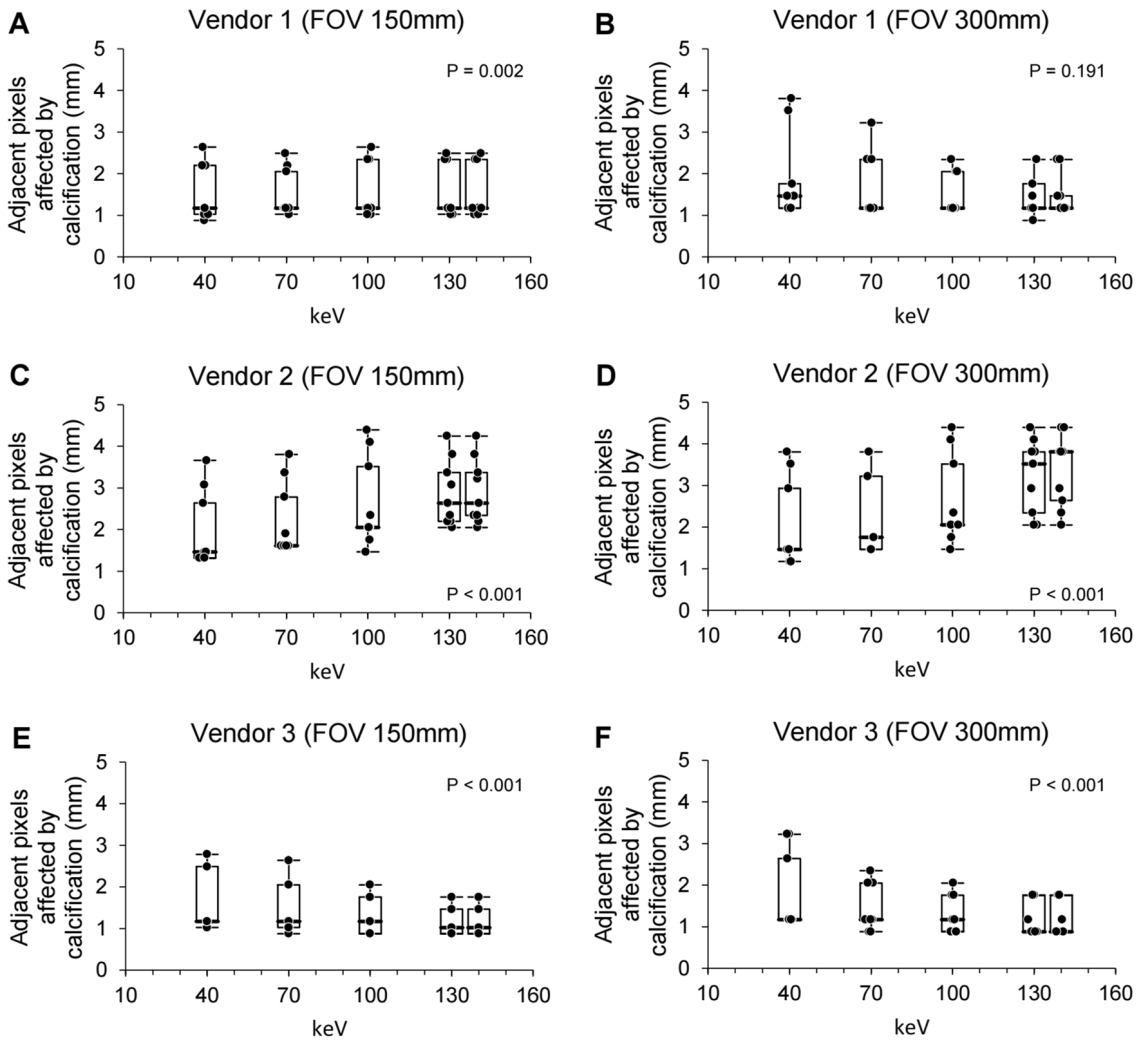
Box plots of adjacent pixels affected by calcification using different kilo-electron volts levels of virtual monochromatic images of a phantom. Box plots of adjacent pixels affected by calcification for different kilo-electron volts levels for small (**A**, **C**, and **E**) or large (**B**, **D**, and **F**) field of view of virtual monochromatic images of a phantom using vendor 1 (**A** and **B**), 2 (**C** and **D**), or 3 (**E** and **F**). The bold horizontal line indicates the median, while the top and bottom horizontal lines of boxes show the quartiles of 25% and 75%, respectively. Upper and lower horizontal lines outside of boxes show the maximum and minimum, respectively. Friedman test was used for the calculation of p-value. FOV, field of view; keV, kilo-electron volts; Vendor 1, Siemens; Vendor 2, Philips; Vendor 3, GE.

Next, the adjacent pixels affected by calcification were calculated using different keV levels of virtual monochromatic images. Using virtual monochromatic images, the adjacent pixels affected by calcification were compared for each keV level, and the results showed different aspects of the adjacent pixels affected by calcification among the different keV levels for each CT vendor (Fig. 5). For vendor 1, different keV levels did not exhibit significant differences in the adjacent pixels affected by calcification in a large FOV (p = 0.191). The adjacent pixels affected by calcification increased and decreased significantly with higher keV levels for vendors 2 and 3, respectively (p < 0.001). The different aspects of quantitative measurements according to keV levels for each CT vendor implied no significant relationship between adjacent pixels affected by calcification and higher X-ray energy levels.

### Clinical study: analysis of adjacent pixels affected by calcification using virtual monochromatic images

**Figure 6:**
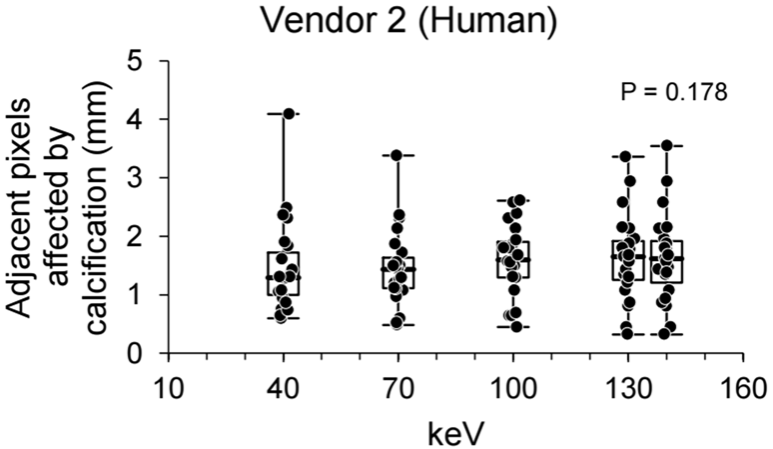
A box plot of adjacent pixels affected by calcification of human virtual monochromatic images. A box plot of adjacent pixels affected by calcification for different kilo-electron volts levels of human virtual monochromatic images using vendor 2. The bold horizontal line indicates the median, while the top and bottom horizontal lines of boxes show the quartiles of 25% and 75%, respectively. Upper and lower horizontal lines outside of boxes show the maximum and minimum, respectively. Friedman test was used for the calculation of p-value. keV, kilo-electron volts; Vendor 2, Philips.

For the clinical study, adjacent pixels affected by calcification were calculated using different keV levels of virtual monochromatic images. Using virtual monochromatic images, adjacent pixels affected by calcification were compared for each keV level (Fig. 6), and the results failed to show significant differences in adjacent pixels affected by calcification among the keV levels for vendor 2 (p = 0.178). Consequently, quantitative measurements showed that higher keV levels failed to significantly reduce the number of adjacent pixels affected by calcification.

## Discussion

Previous studies have suggested that increasing the kVp in conventional polychromatic images or virtual monochromatic images with higher keV levels can reduce blooming artifacts caused by calcification^17–20^. However, we hypothesized that higher X-ray energy levels decrease blooming artifacts caused by calcification with subjective visual assessment in CT images but fail to reduce the number of adjacent pixels affected by calcification with quantitative measurement. Therefore, this study focused on the quantitative analysis of the adjacent pixels affected by calcification using different X-ray energy levels, such as kVp or keV, through phantom and clinical studies. Analysis of adjacent pixels affected by calcification showed no significant relationship between X-ray energy levels.

A higher X-ray energy level induces a decrease in the attenuation values of CT images, which leads to a reduction in blooming artifacts caused by calcification on visual subjective assessment. However, no study has quantitatively measured the adjacent pixels affected by calcification according to different X-ray energy levels. This study quantitatively measured adjacent pixels affected by calcification using a novel method that employs FW3M as the actual calcified length for both phantom and clinical studies. In case of the phantom study, the actual calcified diameter is known, such as 6, 5, or 4 mm, and these values could serve as the ground truth. Although the physical length could have been used as the ground truth in the phantom study, we opted to use FW3M for calculating the actual calcified length in both phantom and clinical studies for consistency, given that the actual calcified length was unknown in the clinical study. We have demonstrated that FW3M is a reliable method, resulting in a mean error of 0.05 mm and a S.E.M of 0.01 mm, for estimating the actual calcified length and was thus used for calculating the ground truth in both phantom and clinical studies.

Quantitative measurements of adjacent pixels affected by calcification showed no relationship with higher X-ray energy levels. This observation can be applied for the improvement and development of a coronary artery segmentation software platform using CT images. For accurate coronary artery segmentation, the exact separation between the lumen and calcified plaque is critical. However, blooming artifacts caused by calcification mainly induce the false detection of lumen boundaries^21^. To improve the segmentation algorithm for the separation of the lumen and calcified plaque, several approaches are necessary, independent of the X-ray energy level, because the segmentation process is related to quantitative measurement but not to visual subjective assessment.

In case of image quality, the results showed no significant change in noise and signal components with increased kVp. There are references indicating no significant difference in signal components with varying kVp levels^22–24^. We believe that the size of the phantom may have contributed to the lack of significant change in noise and signal components. The phantom was designed with dimensions of 100 mm in width, 100 mm in height, and 5 mm in thickness. The size of the phantom can affect image noise, as photon penetration decreases in larger phantoms, requiring higher X-ray beam energy to achieve the same noise level^25^. Due to the small size of the phantom, variation in signal components and noise may have not had a significant impact with different kVp levels. However, in the virtual monochromatic images, we utilized the lowest X-ray energy level at 40 keV. The maximum HU value at 40 keV exponentially decreased with higher keV levels, potentially leading to significant changes in signal components.

The results of the phantom study using different keV levels in virtual monochromatic images showed different aspects of adjacent pixels affected by calcification for each vendor, implying that different approaches are necessary to reduce adjacent pixels affected by calcification in each vendor under the current circumstances. In the future, innovative approaches are necessary to reduce the number of adjacent pixels affected by calcification using quantitative measurements.

There are some limitations in this study. First, phantom and clinical studies differ in their input settings. The CT images in the clinical study contained contrast agent alongside the calcified plaque, whereas the phantom study included only the calcified plaque. Second, the difference in input settings led to the calculation issue of adjacent pixels affected by calcification. In the clinical study, the same calculation method as in the phantom study was applied, which used the FW3M to determine the actual calcified length. This measurement assessed the spread of calcified length over the region of the contrast agent, resulting in adjacent pixels affected by calcification being calculated as zero in the direction of the contrast agent. However, this was observed even in the CT images at all keV levels. Therefore, comparing adjacent pixels affected by calcification at different X-ray energy levels was achievable by using the direction that excluded the contrast agent. Finally, the clinical study was conducted using only vendor 2 owing to data unavailability.

In our study, we utilized virtual monochromatic images, rather than true monochromatic images, to assess adjacent pixels affected by calcification across different X-ray energy levels. However, there is a significant impact of the distinction between true and virtual monochromatic images on the manifestation of blooming artifacts. Given the circumstances, true monochromatic images were unavailable, and only virtual monochromatic images were assessable. In the future studies, we intend to analyze adjacent pixels affected by calcification based on different X-ray energy levels using true monochromatic images.

In summary, the adjacent pixels affected by calcification were quantitatively measured using the brightened length, excluding the actual calcified length. The two-part study showed that changes in X-ray energy levels, such as kVp or keV, in polychromatic or virtual monochromatic images did not exhibit a reduction in adjacent pixels affected by calcification. This study indicated that higher X-ray energy levels, such as kVp or keV, decrease blooming artifacts caused by calcification with subjective visual assessment but fail to reduce adjacent pixels affected by calcification with quantitative measurement.

## Methods

### Phantom study

#### (i) Phantom design

To investigate the adjacent pixels affected by calcification with quantitative measurements, a phantom was designed using Solidworks software (Dassault Systemes). The design considered various geometric differences such as size, shape, or degree of calcification, with dimensions of 100 mm in width, 100 mm in height, and 5 mm in thickness (Fig. 1). The final phantom was manufactured with acrylonitrile–butadiene–styrene using a three-dimensional (3D) printer (3DWOX 2X, Sindoh), and was completely filled with a mixture of calcium phosphate powder and droplets of distilled water. When filling the manufactured phantom with the calcium mixture, a sharp tool was used to remove air bubbles through several small holes at the bottom of the phantom.

#### (ii) Computed tomography protocols and reconstruction

All CT scans were performed as helical scans using an abdominal protocol five times on different dates from June 22, 2022, to August 9, 2022, at Seoul National University Hospital using three different DECT scanners (SOMATOM Force, Siemens [vendor 1]; IQon – Spectral CT, Philips [vendor 2]; Revolution Apex, GE [vendor 3]). Conventional polychromatic scans were performed using three different CT scanners at 80, 100, 120, and 140 kVp at different FOVs (large FOV, 300 mm; small FOV, 150 mm) to analyze the adjacent pixels affected by calcification according to the X-ray energy difference. The tube current was adjusted to 250 mA for each vendor, except for the virtual monochromatic images for vendor 1, which used two tubes for scanning (240 mA for 80 kV and 120 mA for 140 kV). Rotation times of 330–1000 ms, collimation of 192 × 0.6 mm/128 × 0.6 mm/16 × 0.625 mm/40 × 0.625 mm, pitches of 0.6–0.984 were used for scanning parameters for different vendors. CT images were reconstructed using a unique algorithm for each vendor with slice thickness of 0.6–0.8 mm and detailed information is provided in Supplementary Tables S1 and S2. Virtual monochromatic scans were performed for each DECT vendor using different implementation methods ([a] SOMATOM Force, Siemens; dual independent source with detector; [b] IQon—Spectral CT, Philips; single source and dual-layer detector; and [c] Revolution Apex, GE; single source with detector using rapid tube potential switching). Virtual monochromatic reconstruction was performed at 40, 70, 100, 130, and 140 keV using Syngo.via (Siemens), IntelliSpace Portal (Philips), and A/W server 2 (GE).

#### (iii) Measurement of distortion angle of a phantom

To calculate the adjacent pixels affected by calcification accurately, the distortion angle of the phantom was measured for each scan using different vendors (Table 1 and Supplementary Fig. S1). The distortion angle was measured in degrees for the anterior or left views of the reconstructed 3D images of the phantom using Rapidia 3D version 2.8 (INFINITT Co., Ltd.). The distortion angle in the axial view was calculated using the following equation:

$$ Distortion angle \left( {degrees} \right) = \frac{180}{\pi } \times arctan\sqrt {(tan\theta_{1} )^{2} + (tan\theta_{2} )^{2} } $$

where θ_1_ and θ_2_ are the distortion angles in the anterior and left views, respectively. The measurement error according to the distortion angle was calculated using the equation as follows: $Measurement error \left( \% \right) = \left( {1 - \frac{1}{\cos \theta }} \right) \times 100$.

where θ is the calculated distortion angle.

### Clinical study

The Institutional Review Board of Seoul National University Hospital (IRB # 2308-169-1461) approved the clinical study protocol of this retrospective study and waived the requirement for informed consent. All experiments and methods were performed in accordance with relevant guidelines and regulations.

#### (i) Study population

Patients who underwent DECT between June 2019 and May 2020 at Seoul National University Hospital with virtual monochromatic reconstruction were included in this study. Patients without calcifications were excluded. Overall, 23 calcified plaques from 10 patients (men, 60%; mean age, 66.9 years; range, 29–86 years) were included in the analysis.

#### (ii) Computed tomography protocols and reconstruction

All CT scans were performed using a DECT scanner (IQon—Spectral CT, Philips). A tube voltage of 120 kVp, tube currents of 42–134 mA, rotation times of 270–330 ms, collimation of 40 × 0.625 mm, a pitch of 0.18 were used for scanning parameters. Polychromatic images were reconstructed using the IMR1 algorithm with slice thickness of 0.8 mm. Virtual monochromatic reconstruction was performed at 40, 70, 100, 130, and 140 keV using IntelliSpace Portal (Philips).

### Measurement of contrast-to-noise ratio, signal-to-noise ratio, and noise

To validate the quality of the CT images, the CNR, SNR, and noise were calculated for the polychromatic and virtual monochromatic images using each vendor. The region of interest (ROI) was set as the calcium region, and the background region was located at the lower and right parts of the ROI for the phantom and clinical study, respectively (Fig. 1). The CNR was defined using the following formula:

$$ CNR = \frac{{\overline{HU}_{ROI} - \overline{HU}_{backgrounud} }}{{SD of HU_{background} }} $$

representing the difference between the average HU in the ROI and the background region, divided by the standard deviation (SD) of the HU in the background region. The SNR was defined using the following formula:

$$ SNR = \frac{{\overline{HU}_{ROI} }}{{SD of HU_{background} }} $$

representing the average HU of the ROI divided by the SD of the HU in the background region. Noise was defined using the following formula:

$$ Noise = SD of HU_{background} $$

representing the SD of the HU in the background region. All calculations were performed using Matlab®.

### Measurement of Adjacent Pixels Affected by Calcification

Adjacent pixels affected by calcification were defined using the following formula:

$$ Adjacent pixels affected by calcification = length_{brightened} - length_{actual calcification} $$

representing the brightened length excluding the actual calcified length. For both phantom and clinical studies, the actual calcified length was determined using the definition of the FW3M instead of the FWHM, which is a widely used measurement method^26^. A third and half maximum attenuation value of the calcified length with a background value of zero was used for the FW3M and FWHM measurements, respectively. Calcifications of different sizes with concentric shapes were used for the FW3M and FWHM measurements, and the mean error against the real diameter was calculated.

The maximum and minimum thresholds of the brightened length were determined as the attenuation values located at the edge of the FW3M and at 5% of the highest brightness of the calcification, respectively (Supplementary Fig. S2). For different shapes of calcification, such as round, eccentric, and stent-like shapes, we employed a consistent method to measure adjacent pixels affected by calcification. This involved determining the center point of each calcification shape and averaging the pixel spacing of the brightened lengths in four directions (top, bottom, left, and right) from this center point. All calculations were performed using Matlab®.

### Statistical analysis

Adjacent pixels affected by calcification were investigated using nonparametric tests to analyze the effects of different kVp or keV levels using CT images. For the phantom study, all statistical analyses were performed using the median value obtained from five repeated CT scans. The Wilcoxon signed-rank test was used to compare the mean error for estimating the actual calcified length using the FWHM or FW3M with a reference of 0.04 mm^27^. The Friedman test was used to compare (i) CT image quality among different kVp or keV levels using CNR, SNR, and noise and (ii) adjacent pixels affected by calcification of different kVp or keV levels^28^. Statistical significance was set at p < 0.05. All analyses were conducted using R version 4.2.0.

### Supplementary Information

Supplementary Information.

## Data Availability

The data set analyzed during the current study are not publicly available due to medical confidentiality but are available from the first author on reasonable request summarized form pending the approval of the IRB.
