# Supplementary material for: Quantitative analysis of blooming artifact caused by calcification based on X-ray energy difference using computed tomography
Source: Sci Rep. 2024 May 21;14:11539. doi: 10.1038/s41598-024-61187-z (PMC11109228; doi:10.1038/s41598-024-61187-z)
Supplement: Supplementary file 1 — Supplementary Information. [file 41598_2024_61187_MOESM1_ESM.docx]

**Supplementary Information**

**Quantitative analysis of blooming artifact caused by calcification based on X-ray energy difference using computed tomography**

**Daebeom Park^4^, Eun-Ah Park^1,2^, Baren Jeong^1^, Yoon Seong Lee^1^, and Whal Lee^1,2,3,4^**

^1^ Department of Radiology, Seoul National University Hospital, Seoul, Korea.

^2^ Department of Radiology, Seoul National University College of Medicine, Seoul, Korea.

^3^ Institute of Radiation Medicine, Seoul National University Medical Research Center, Seoul, Korea.

^4^ Department of Clinical Medical Sciences, Seoul National University College of Medicine, Seoul, Korea.

**Corresponding Author:** Whal Lee, MD, PhD, Department of Radiology, Seoul National University Hospital, 101 Daehak-ro, Jongno-gu, Seoul 03080, Korea. Tel: (822) 2072-0898, Fax: (822) 743-6385, E-mail: [whal.lee@gmail.com](mailto:whal.lee@gmail.com)

**Contents**

**I. Supplementary Figures**

Supplementary Figure S1. Measurement of distortion angle of a phantom.

Supplementary Figure S2. Computed tomography image of calcification with indicated boundaries.

**II. Supplementary Tables**

Supplementry Table S1. Computed tomography parameters for different vendors of the phantom study.

Supplementry Table S2. Volume computed tomography dose index for different vendors.

**I. Supplementary Figures**

**Supplementary Figure S1.** **Measurement of distortion angle of a phantom.**

The distortion angle in the (A) anterior or (B) left views of the phantom. Three-dimensional images of the phantom were used to measure the distortion angle in degrees.

**Supplementary Figure S2.** **Computed tomography image of calcification with indicated boundaries.**

The red and yellow dotted lines indicate the actual calcified boundary, determined by the full width at third maximum, and the brightened boundary, calculated as 5% of the maximum brightness of the calcification, respectively.

**II. Supplementary Tables**

**Supplementary Table S1. Computed tomography parameters for different vendors of the phantom study.**

|  | Polychromatic image | | | Virtual monochromatic image | | |
| --- | --- | --- | --- | --- | --- | --- |
|  | Vendor 1 | Vendor 2 | Vendor 3 | Vendor 1 | Vendor 2 | Vendor 3 |
| kVp | 80, 100,  120, 140 | 80, 100,  120, 140 | 80, 100,  120, 140 | 80 / 140  (dual source) | 140  (single source) | 80 / 140  (fast switching) |
| keV | NA | NA | NA | 40, 70, 100, 130, 140 | 40, 70, 100, 130, 140 | 40, 70, 100, 130, 140 |
| Field of view (mm) | 150, 300 | 150, 300 | 150, 300 | 150, 300 | 150, 300 | 150, 300 |
| Tube current (mA) | 250 | 250 | 250 | 240 / 120 | 250 | 250 |
| Scan type | Helical (Abdomen) | Helical (Abdomen) | Helical (Abdomen) | Helical (Abdomen) | Helical (Abdomen) | Helical (Abdomen) |
| Rotation time (sec) | 0.5 | 0.33 | 1 | 0.5 | 0.33 | 1 |
| Collimation (mm) | 192 × 0.6 | 16 × 0.625 | 40 × 0.625 | 128 × 0.6 | 16 × 0.625 | 40 × 0.625 |
| Pitch | 0.6: 1 | 0.799: 1 | 0.984: 1 | 0.6: 1 | 0.799: 1 | 0.984: 1 |
| Slice thickness (mm) | 0.6 | 0.8 | 0.625 | 0.6 | 0.8 | 0.625 |
| Reconstruction algorithm | Br40 | IMR1 | Standard | Qr40 | Spectral | Standard |

keV = kilo-electron volts; kVp = kilovoltage peak; NA = not applicable; Vendor 1 = Siemens; Vendor 2 = Philips; Vendor 3 = GE

**Supplementary Table S2. Volume computed tomography dose index for different vendors.**

|  | CTDIvol (mGy) | | |
| --- | --- | --- | --- |
|  | Vendor 1 | Vendor 2 | Vendor 3 |
| kVp 80 | 4.79 | 8.50 | 3.31 |
| kVp 100 | 10.01 | 16.30 | 6.49 |
| kVp 120 | 16.69 | 25.70 | 10.48 |
| kVp 140 | 24.34 | 37.00 | 15.26 |
| keV | 17.16 | 37.00 | 8.34 |

CTDIvol = volume computed tomography does index; keV = kilo-electron volts; kVp = kilovolatge peak; Vendor 1 = Siemens; Vendor 2 = Philips; Vendor 3 = GE
